# Ucp2 Induced by Natural Birth Regulates Neuronal Differentiation of the Hippocampus and Related Adult Behavior

**DOI:** 10.1371/journal.pone.0042911

**Published:** 2012-08-08

**Authors:** Julia Simon-Areces, Marcelo O. Dietrich, Gretchen Hermes, Luis Miguel Garcia-Segura, Maria-Angeles Arevalo, Tamas L. Horvath

**Affiliations:** 1 Instituto Cajal, CSIC, Madrid, Spain; 2 Program in Integrative Cell Signaling and Neurobiology of Metabolism, Section of Comparative Medicine, Yale University School of Medicine, New Haven, Connecticut, United States of America; 3 Department of Biochemistry, Universidade Federal do Rio Grande do Sul, Porto Alegre, Brazil; 4 Departments of Neurobiology and Obstetrics, Gynecology and Reproductive Sciences, Yale University School of Medicine, New Haven, Connecticut, United States of America; Case Western Reserve University, United States of America

## Abstract

Mitochondrial uncoupling protein 2 (UCP2) is induced by cellular stress and is involved in regulation of fuel utilization, mitochondrial bioenergetics, cell proliferation, neuroprotection and synaptogenesis in the adult brain. Here we show that natural birth in mice triggers UCP2 expression in hippocampal neurons. Chemical inhibition or genetic ablation of UCP2 lead to diminished neuronal number and size, dendritic growth and synaptogenezis in vitro and impaired complex behaviors in the adult. These data reveal a critical role for *Ucp2* expression in the development of hippocampal neurons and circuits and hippocampus-related adult behaviors.

## Introduction

The perinatal environment represents a critical period in brain development that determines the adult architecture of the central nervous system and related functions. In vitro approaches to study aspects of neuronal differentiation and synaptogenesis have long been used to gain a better understanding of the developmental processes of neuronal circuits. However, practical aspects of the successful maintenance of primary neuronal cultures do not represent close mimicking of the in vivo environment of neurons during the perinatal period. Specifically, the nutrient composition of culture media is vastly different from that provided by the placenta, clostrum and breast milk, the main source of fuel for developing neurons perinatally. An important characteristic of breast milk, in contrast to placental blood support, is its high content of long chain fatty acids besides glucose [1]. We have identified mitochondrial uncoupling protein 2 (*Ucp2*) as a critical determinant of fatty acid utilization by adult neurons [2]. *Ucp2* promotes free radical scavenging [3]–[5], which is critical for enabling fatty acid beta oxidation in neurons [2]. This mechanism is also critical for adult synaptogenesis [6]. Ucp2 is also implicated in protection of adult [7] as well as developing neurons in a febrile seizure model in rats at a time of breastfeeding [8]. In the present study, we sought to determine whether *Ucp2* induction occurs in the hippocampus perinatally, and if so, whether Ucp2-associated cellular mechanisms are involved in the development of neuronal circuits *in vitro* with implications for adult behavior.

## Methods

### Animals

CD1 mice were raised in the Cajal Institute and all procedures for handling and killing the animals used in this study were in accordance with the European Commission guidelines (86/609/CEE) and were approved by the animal care and use committee of the Cajal Institute. Vaginal birth occurs at around E18 in the CD1 mouse colony of the animal facility of the Cajal Institute. *Ucp2* gene knockout and wild type littermates C57BL6 mice were generated as described previously (20) All procedures were approved by the Institutional Animal Care and Use Committee of Yale University.

### Hippocampal Neuronal Cultures and Incubation Conditions

The hippocampus was dissected out from embryonic day 18 (E18) mouse embryos and dissociated to single cells after digestion with trypsin (Worthington Biochemicals, Freehold, NJ) and DNase I (Sigma-Aldrich). Neurons were plated on 6-wells plates or glass coverslips coated with poly-L-lysine (Sigma-Aldrich) at a density of 200–600 neurons/mm^2^, and they were cultured in Neurobasal supplemented with B-27 and GlutaMAX I (Invitrogen, Crewe, United Kingdom). Under the conditions used, our cultures were nearly devoid of glia. After the indicated time in vitro, the cultures were fixed for immunostaining (4% paraformaldehyde/4% sucrose in PBS) or harvested for real-time PCR. Parallel cultures were prepared by adding to the cultures medium 20 µM genipin (Wako Chemicals USA) before plating cells.

### Quantitative real time polymerase chain reaction (real time PCR)

Total RNA was extracted from cultures at different stages of neuron development with illustra RNAspin Mini RNA isolation kit from GE Healthcare (Buckinghamshire, UK). On the other hand, hippocampi were dissected from E18, delivered with Caesarian section (CS) or vaginal birth (VB), P10 and adult mouse, disrupted and homogenized in lysis buffer and total RNA extracted with the same kit. First strand cDNA was prepared from 2 µg RNA using the RevertAid™ H Minus First Strand cDNA Synthesis Kit (MBI Fermentas, Bath, UK) according to the supplied protocol. After reverse transcription, the cDNA was diluted 1∶3 and 5 µl were amplified by real-time PCR in 20 µl using SYBR Green Master Mix or TaqMan Universal PCR Master Mix (Applied Biosystems, AB, Foster City, CA) in a ABI Prism 7000 Sequence Detector (AB), with conventional AB cycling parameters (40 cycles of 95°C, 15 s, 60°C 1 min). Primer sequences were designed using Primer Express (AB) and were for *Ucp2*: forward, 5′-ACAAGACCATTGCACGAGAG-3′ and reverse, 5′-ATGAGGTTGGCTTTCAGGAG-3′; for *Nrf1*: forward, 5′-CGCAGCACCTTTGGAGAA-3′ and reverse, 5′-CCCGACCTGTGGAATACTTG-3′; for *Tfam*: forward, 5′-GGAATGTGGAGCGTGCTAAAA-3′ and reverse, 5′-TGCTGGAAAAACACTTCGGAATA-3′. *Ngn3* and Glyceraldehyde 3-phosphate dehydrogenase (*Gapdh*), which was selected as control housekeeping gene, were analyzed using Assay-on-Demand gene expression products (AB). After amplification, a denaturing curve was performed to ensure the presence of unique amplification products. For visualizing and sequencing the PCR products, each mixture was electrophoresed in 2% (w/v) ethidium bromide-stained agarose gels. Then, bands were excised and cDNA was purified using the QIAquick PCR purification Kit (Qiagen, GmbH, Germany). One hundred nanograms of each sample were sequenced (Automatic Sequencing Center, CSIC, Madrid, Spain) with the corresponding forward or reverse primer. The obtained sequence was aligned with the expected sequence of each transcript obtained from the GenBank. All reactions were performed in triplicate and the quantities of target gene expression were normalized to the corresponding *Gapdh* expression in test samples and plotted.

### Western blot analysis of UCP2 protein expression

Lysate from hippocampal samples of animals at the time of birth with VB, CS or at postnatal day 10 (born naturally) or adulthood (born naturally) were processed for Western blot analyzes using UCP2 antisera and procedures as described in Horvath et al. 2002 [9]. Mouse hippocampi were homogenized in 20 mM Tris/HCl (pH 7.4), 10 mM potasium acetate, 1 mM DTT, 1 mM EDTA, 0,25% NP-40 and an anti-protease cocktail (Roche Diagnostics) and centrifuged at 700 g (10 min). The supernatant was then centrifuged at 10 000 g (15 min), and the pellets were resuspended in SDS-PAGE loading buffer.

Proteins were resolved by SDS-PAGE and transferred onto nitrocellulose membranes (Millipore). The membranes were blocked in Tris-buffered saline containing 0.1% Tween 20 and 2% ECL advance blocking reagent (Amersham) and incubated first with rabbit anti-UCP2 polyclonal antibody (1∶2000) [9] and rabbit anti-aralar antibody (loading control, 1∶1000; a gift from doctor Araceli del Arco) and then with horseradish peroxidase-conjugated goat anti-rabbit and goat anti-mouse secondary antibodies (1∶10000; Jackson Immuno Research). Specific proteins were visualized with enhanced chemiluminescence detection reagent according to the manufacturer's instructions (Amersham). Densitometry and quantification of the bands were carried out using the Quantity One software (Bio-Rad). Statistical analysis of the data was performed using an unpaired t-test.

### Immunocytochemistry, image acquisition and morphometric analysis

The following primary antibodies were used: chicken anti-βIII tubulin (1∶1000; Abcam, Cambridge, UK) and mouse anti-synaptophysin I (1∶500; Progen, Heidelberg, Germany). To verify that the labeling was caused specifically by the primary antibodies, it was either omitted or replaced by similarly diluted normal serum from the same species. Secondary antibodies were donkey anti-chicken-FITC (1∶500) and goat anti-mouse-Cy3 (1∶1000), both from Jackson Immuno Research (West Grove, PA). For the evaluation of soma size, dendritic morphology and presynaptic terminal identification in dissociated cell cultures, labeled neurons were visualized by standard epifluorescence under a 40× oil objective under a Leica microscope. Images were captured with a Leica digital camera controlled by the Leica software (Leica, Heidelberg, Germany). Somas size was evaluated using ImageJ 1.38 (NIH). Primary dendrite number i.e., the number of dendrites associated with the soma, and terminal counts were performed manually. A circular region of interest (ROI) with a diameter of 100 µm was projected onto the βIII- Tubulin labeled neuron, its center roughly coinciding with the center of the soma. Synaptic terminals contacting somata or dendrites were counted within the circular ROI.

To determine the number of neurons in the cultures, at least fifteen culture fields of 600 µm^2^ were photographed per stage using a reverse microscope equipped with phase contrast, at 1.5 hours after seeding and at the time corresponding to different stage of neuron development. The number of cell was counted manually.

To identify neuronal cells, rabbit anti-Tau (1∶20; Abcam, Cambridge, UK) as axonal marker and mouse anti-MAP2 (1∶500; Sigma-Aldrich) as dendrite marker were used. Secondary antibodies: goat anti-rabbit-Alexa488 (1∶500) and goat anti-mouse-Cy3 (1∶1000), both from Jackson Immuno Research (West Grove, PA).

### Open-Field Testing

The open field test apparatus was a square, polyurethane arena (36.5 cm×36.5 cm×30 cm, Plexiglas). The animal was placed in corner of the apparatus locomotion speed, distance traveled, entries into the central zone, and time spent in contact with the outer walls, were recorded for 5 minutes. Behavioral testing took place from 1000 to 1400 h (i.e. in the light phase of the light-dark cycle). The apparatus was cleaned with 10% ethanol after each animal exposure. ANY-Maze Software™ (Stoelting Company, Wood Dale, IL) was used to record and analyze behavioral data.

### Y-Maze Testing

Spatial memory was assessed using the two-trial Y-maze task. A single Y-maze was made of black Plexiglas and consisted of three arms with an angle of 120° between each of the two arms. Each arm was 8 cm×30 cm×15 cm (width×length×height). The three arms were randomly designated: start arm, in which the mouse started to explore (always open), novel arm, in which the mouse started to explore (always open), novel arm, which was blocked during the first trial but open during the second trial.

The maze was placed on a flat surface within the behavioral testing room. Proximal visual cues (pictures within the arms of the apparatus) and distal visual cues (the configuration of the room, curtain, wall art) remained constant throughout testing. The floor of the maze was covered with white chip bedding. Between each trial the apparatus was cleaned with 10% ethanol and new bedding was added. Behavioral testing took place from 1000 to 1400 h (i.e. in the light phase of the light-dark cycle).

The Y-maze test consisted of two trials separated by an inter-trial interval (ITI) of 60 minutes to assess spatial memory. The first trial had a five-minute duration and allowed the mouse to freely explore only two arms (start arm and other arm) while the third arm was blocked. After a 60 min ITI, the second trial also of five minutes duration was conducted during which all three arms were accessible and novelty vs. familiarity was compared in all three arms. ANY-Maze Software™ (Stoelting Company, Wood Dale, IL) was used to record and analyze behavioral data.

### Statistical Analysis

All data are expressed as the mean + SEM or mean ± SEM from at least 3 experiments. Data was analyzed using the Graph Pad Prism 5.0 program (GraphPad Software, Inc., San Diego, CA). The means between two samples were analyzed by student t-test and between more than two samples by one-way ANOVA followed by Bonferroni post hoc. Significance was taken at p<0.05.

## Results

### UCP2 mRNA is induced by birth

**Figure 1:**
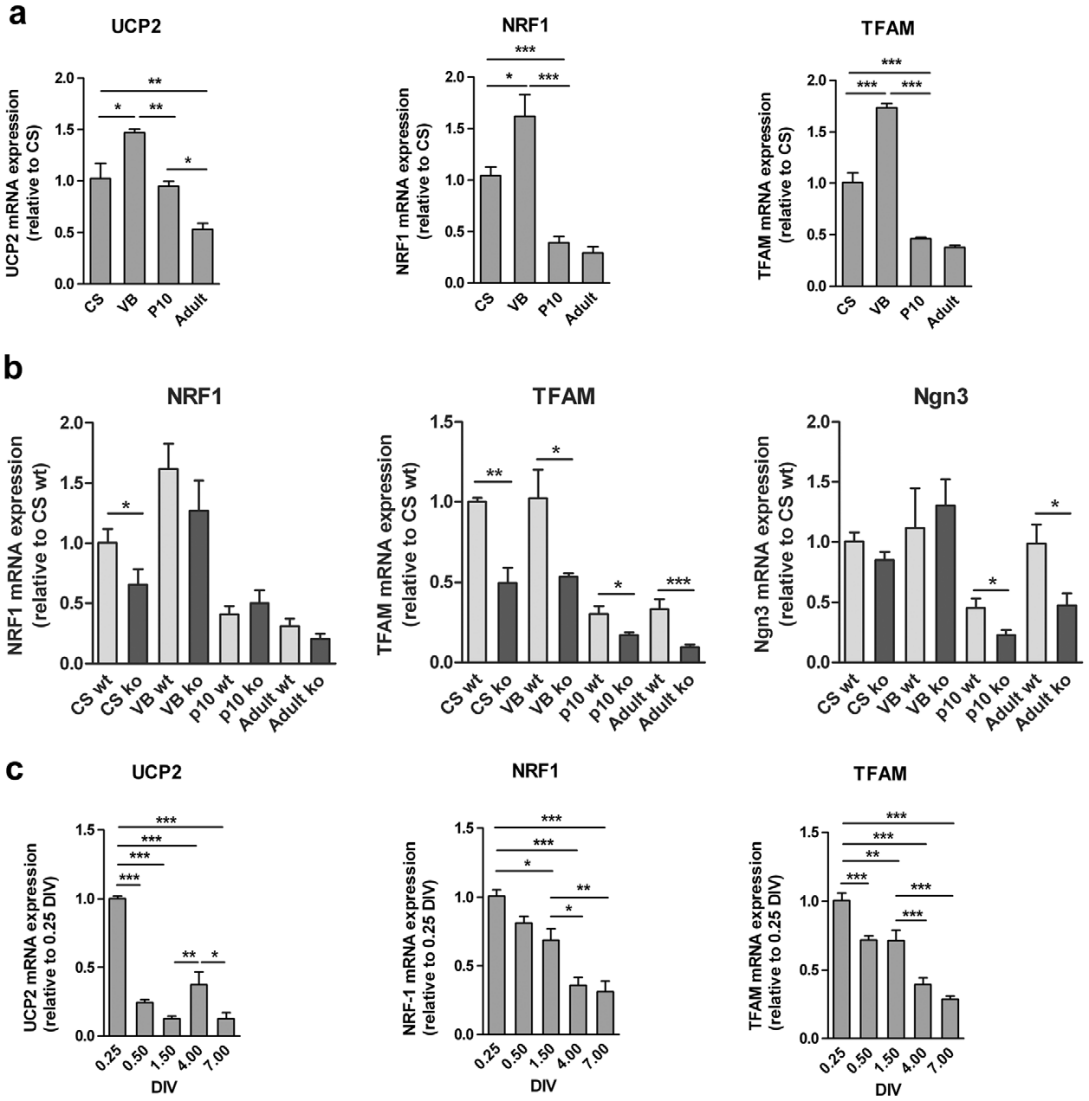
Time course changes in *Ucp2*, *Nrf1* and *Tfam* mRNA expression in the mouse hippocampal development. (**a and c**) Total RNAs were extracted from the hippocampal tissue (**a**) or cultured hippocampal neurons (**c**), retrotranscribed to cDNA and specific mRNA expression of each gene analyzed by real time PCR (see supplementary material for methods). *Gapdh* gene expression was used as internal standard. Data represent the mean+SEM (n = 4 for each). ANOVA revealed significant differences among experimental groups (**a**): *Ucp2*: F_3,11_ = 20.96; p<0.0001; *Nrf1*: F_3,45_ = 28.64; p<0.0001; *Tfam*: F_3,11_ = 202.5; p<0.0001; (**c**): *Ucp2*: F_5,72_ = 61.79; p<0.0001; *Nrf1*: F_5,34_ = 20.42; p<0.0001; *Tfam*: F_5,34_ = 39.35; p<0.0001. (**b**) Total RNAs were extracted from the hippocampal tissue from wild type and *Ucp2* KO mice, retrotranscribed to cDNA and specific mRNA expression of each gene analyzed by real time PCR. *Gapdh* gene expression was used as internal standard. Data represent the mean+SEM (n = 4 for each) and are expressed as relative of the wild type Caesarea delivered fetal value. Asterisks indicate statistical differences between the data sets connected by horizontal lines (*, p<0.05; **, p<0.01; ***, p<0.001), as determined by using the Student's t-test. CS, Caesarian section; VB, vaginal birth; G, genipin.

We analyzed *Ucp2* expression in the hippocampus of mice delivered with Caesarian section (CS), vaginal birth (VB) and in various postnatal ages from animals born naturally. *Ucp2* mRNA levels were induced in newborns by vaginal birth compared to age-matched mice that were delivered with Cesarean section (Fig. 1a). Subsequently, *Ucp2* mRNA levels began to decrease and reached levels below the fetal ones. *Ucp2* related genes involved in mitochondrial biogenesis, *Nrf1* and *Tfam*, showed a similar expression profile (Fig. 1a). Differences in the level of the expression of these latter genes were observed between *Ucp2* KO mice and their wild type littermates in each stage of development (Fig. 1b). *Nrf1* mRNA expression level was decreased in the *Ucp2* KO mice compared to wild type mice at E18 delivered with Caesarian section; *Tfam* mRNA expression level was smaller in the *Ucp2* KO mice than in wild type mice at all developmental stages studied. The expression of *Ngn3* mRNA, a proneural transcription factor related with hippocampal neuron development [10] was decreased in *Ucp2* KO mice compared to wild type mice at P10 and in adults.

### UCP2 mRNA expression is induced by culture conditions

We next used cultured hippocampal neurons from day E18 that show a transition through a sequence of five morphological stages [11]. The level of *Ucp2* mRNA was the highest at stage 1 (Fig. 1c), which corresponds to in vivo stage of birth. Levels of *Ucp2* mRNA decreased at stages of neuron differentiation (2 and 3) and showed a small increase at stage 4, when the dendrites have already been defined and neurons start to establish its synaptic contacts. *Nrf1* and *Tfam* mRNA levels showed shifts in expression that was not always similar to changes in *Ucp2* (Fig. 1c). Thus, in vitro culturing of neurons taken from mice that were surgically delivered has resemblance to the mimics the effect of vaginal delivery on *Ucp2* expression.

### UCP2 protein expression

**Figure 2:**
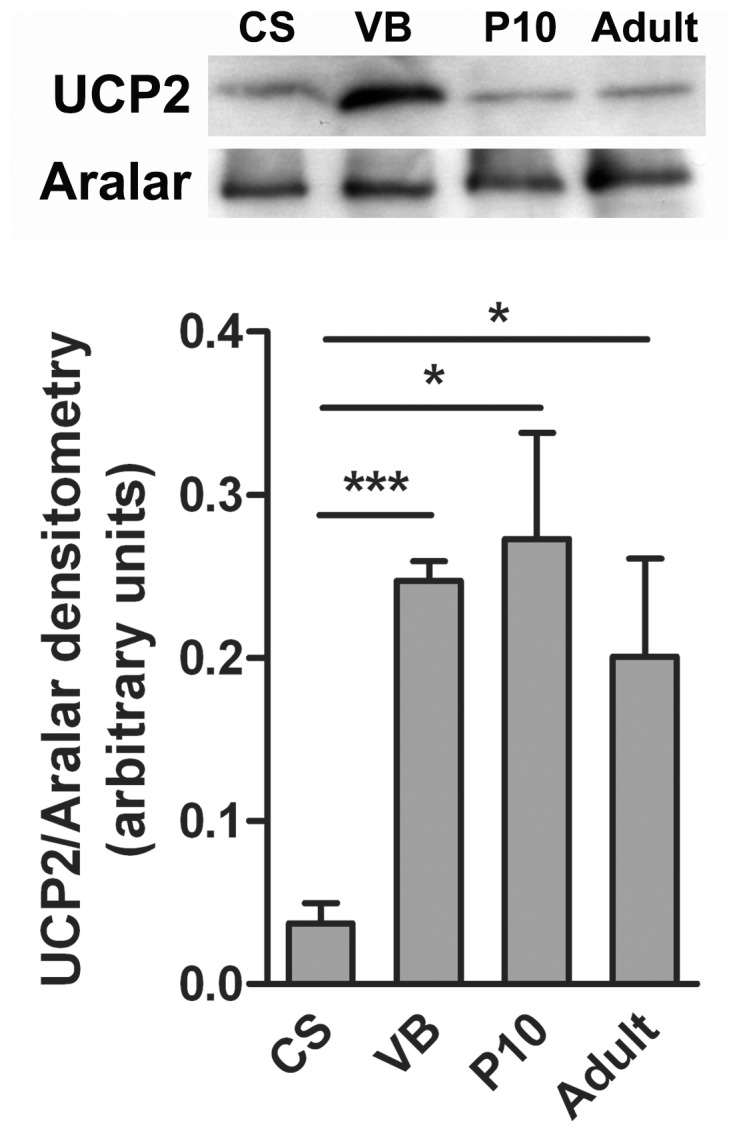
UCP2 protein levels at E18, delivered with Caesarian section (CS) or vaginal birth (VB), P10 and adult mice. Mouse hippocampi were homogenized and mitochondrial proteins were purified and separated by SDS-PAGE. Upper panel, representative Western blot for UCP2 and Aralar (loading control). Bottom panel, graph representing the mean + SEM of densitometric signal of UCP2 versus Aralar protein. The levels of significance were denoted as *p<0.05, ***p<0.001 for the data sets (n = 3) connected by horizontal lines as determined by an unpaired t-test.

We observed a significantly higher level of UCP2 protein expression at the day of delivery in animals that were born via VB compared to those with CS (Fig. 2). In naturally born mice, UCP2 protein remained elevated early post-nataly (P10) as well as in adulthood (Fig. 2).

### Effect of chemical or genetic suppression of UCP2 activity on hippocampal cultures

**Figure 3:**
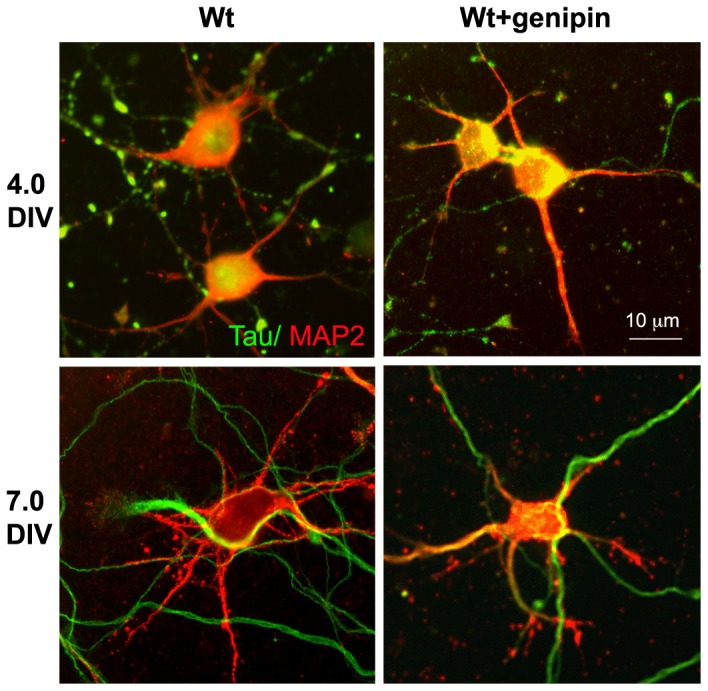
Cultured hippocampal neurons. Cultured hippocampal neurons from wild type mice were treated or untreated with genipin (20 µM). Cells were fixed at 4 DIV and at 7 DIV and immunostained with antibodies against MAP2 (red) and Tau (green) to label dendrites and axons, respectively.

**Figure 4:**
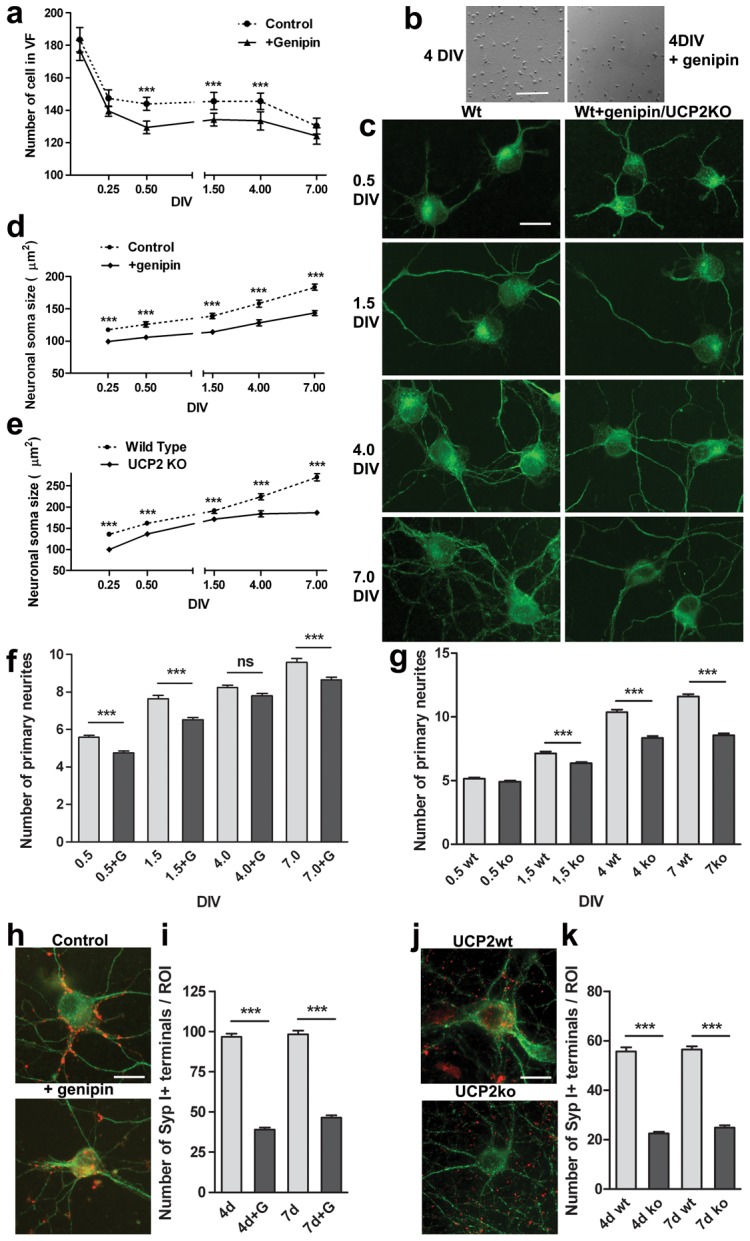
Effect of Ucp2 inhibition and *Ucp2* genetic deletion on morphology and synaptic inputs on cultured hippocampal neurons. (**a**) Cultures were untreated or treated whit genipin (20 µM) and images were taken under a reverse microscope equipped with phase contrast (see supplementary material for methods). Graph represents the number of cell per field at different days in vitro (DIV). (**b**) Representative images of neurons at 4 DIV. Scale bar 100 µm. (**c**) Immunofluorescence images of neurons at different stages of development marked with anti βIII-tubulin antibody. Scale bar 10 µm. (**d,e**) Graphs representing the quantitative analysis of neuronal soma size in different preparations. (**f,g**) Histograms representing the number of primary neurites. (**h,j**) Representative immunofluorescence images of cultured hippocampal neurons fixed at 4 DIV immunostained with antibodies against βIII-tubulin (green) and synaptophysin (red). (**i, k**) Counts of synaptophysin immunoreactive terminals in contact with a neuron per ROI. ROI diameter: 100 µm. Typically 60–75 neurons were evaluated in each condition ((n = 3). Bar represents the mean ± SEM. The level of significance was denoted as ***p<0.001 *p<0.05, **p<0.01 for the data sets connected by horizontal lines as determined by using the Student's t-test.

To test the effect of *Ucp2* induction on neuronal differentiation, cultures first were treated with the Ucp2 inhibitor, genipin [12] (Fig. 3). We have extensively analyzed the effect of genipin on mitochoindrial functions and how they relate to UCP2's effect [2]. In those studies, we confirmed genipin's brain effects but we also showed that genipin has a more broad action on mitochondrial metabolism [2]. Thus, we concluded that while genipin is not UCP2-specific, it does have an overall effect on mitochoindrial and cellular function that is consistent with an effect that opposes UCP2 action. Between 0.5 and 4 DIV, the number of neurons was significantly decreased by genipin treatment compared to control cultures (Fig. 3). Furthermore, genipin caused a decrease of the neuronal soma size, the number of primary neurites and dendrites and the number of synaptophysin positive presynaptic clusters on the soma and dendrites (Fig. 4). Next we analyzed, cultures from *Ucp2* KO mice and their wild type littermates. Similar to the effect of chemical inhibition of UCP2, *Ucp2* KO cultures showed decreased neuronal soma size, decreased number of primary neurites and decreased number of synaptophysin positive terminals compared to the cultures from their wild type littermates (Fig. 4).

### Behavioral tests

**Figure 5:**
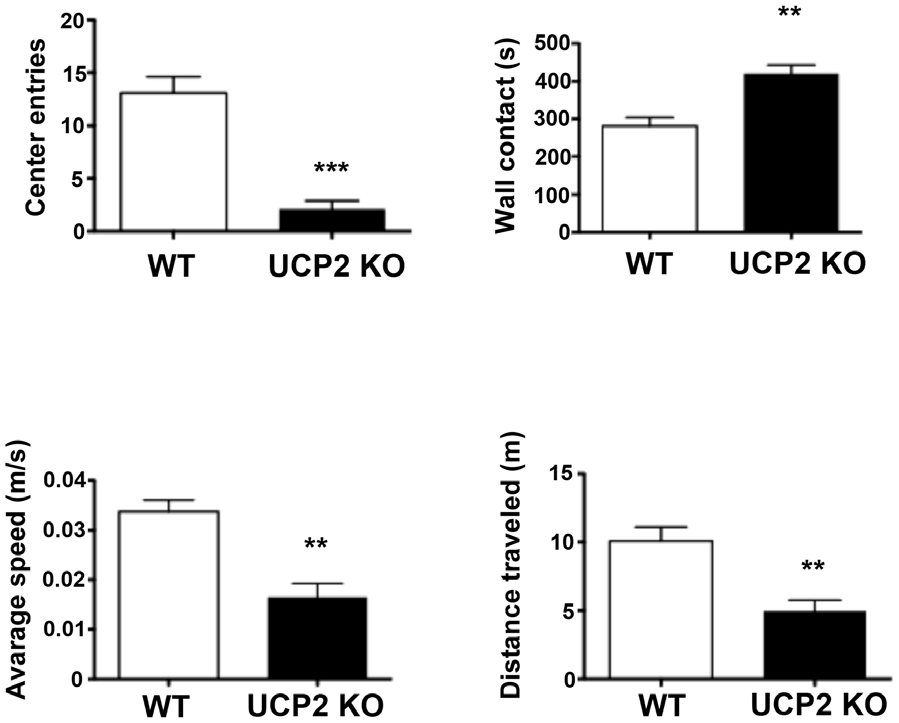
Open field studies. Open field studies revealed significant differences across genotype; ucp2^−/−^ mice were significantly more anxious with fewer entries into the center of the apparatus, greater time in contact with walls of the apparatus and shorter distance travelled. As has been reported elsewhere, ucp2^−/−^ mice were significantly slower than controls. (** p<0.001; *** p<0.0001).

Across a test of unconditioned response to potential threat, ucp2^−/−^ mice displayed significantly more thigmotaxis, fewer trips to the center of the testing arena, shorter distance travelled and a slower rate of locomotion. (Fig. 5a–e).

### Open Field Testing (Fig. 5)

WT, N = 12; KO N = 10. In this measure of exploration stress, ucp2^−/−^ mice travelled significantly less distance that WT (KO distance, 4.9±0.8 m; WT distance,10±1.0 m; t(22) = 4.0, p<0.001). As compared to WT mice, ucp2^−/−^ made significantly fewer trips to the center of the arena (KO trips to center = 2.1±0.80; WT trips to center = 13.08±1.54, t(16) = 6.34, p<0.0001). Time spent in contact with the walls of the apparatus was also significantly different across genotype; ucp2^−/−^ mice displayed high levels of thigmotaxis relative to wild type animals (KO contact with wall = 419.7±23 s; WT contact with wall = 281.4±22.51 s, t(19) = 4.3, p<0.001). As previously reported, ucp2^−/−^ mice were significantly slower than age-matched WT controls (KO average speed m/s = 0.017±0.003; WT average speed m/s = 0.034±0.002, t(22) = 4.8, p<0.0001).

### Spatial Memory Task (Fig. 6)

**Figure 6:**
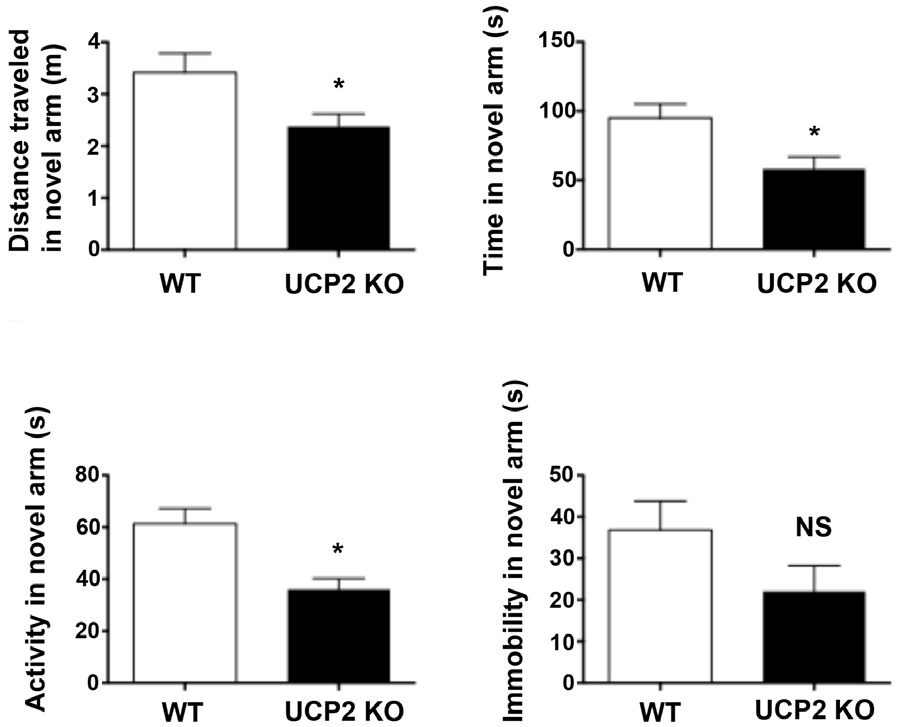
Spatial memory task. In an assessment of hippocampal-based spatial memory, ucp2^−/−^ mice demonstrated an impairment relative to controls with reduced travel distance and time spent in the novel arm.

The distance traveled in the novel arm, a proxy for exploratory behavior related to recognition of novelty differed significantly (KO travel in novel arm 2.38±0.24 m; WT travel in novel arm 3.42±0.37 m, t(11) = 2.36, p<0. 04). Time spent in the novel arm also differed (KO time in novel arm 58.34±8.60 s; WT time in novel arm 95.08±10.14 s, t(12) = 2.76, p<0. 02).

## Discussion

In adult mice, under conditions of synaptogenesis, such as triggered by voluntary exercise, the absence of Ucp2 not only blocks synaptogenesis, but decreases spine synapse number on granule cells as well as on CA1 pyramidal neurons [6]. The role of UCP2 in promotion of scavenging of reactive oxygen species (ROS) in neurons [2], [5], [13] is a likely contributor for the promotion of dendritic growth and synaptogenezis: ROS were found to be a critical negative regulator of hippocampal circuit development [14] and, they are important contributors to neuronal lipid metabolism [2] a process important for membrane fluidity.

The induction of *Ucp2* mRNA by natural birth is consistent with previous findings that correlated the beneficial effects of Ucp2 and high fat content of breast milk with protection of febrile seizures in early postnatal animals [8]. It is reasonable to suggest that *Ucp2* mRNA induction may be associated with hypoxia/ischemia that accompanies vaginal birth. An intracellular mechanism that is known to be activated by hypoxia/ischemia is AMPK [15], which is an upstream inducer of *Ucp2* induction in neurons [2]. AMPK activation suppresses acetyl CoA carboxylase (ACC) activity eliminating the inhibitory effect of malonyl-CoA on carnitine palmitoyl transferase 1 (CPT1) activity. CPT1 activation enhances long chain fatty acid oxidation, which leads to generation of reactive oxygen species (ROS). ROS together with long chain fatty acid availability promotes *Ucp2* transcription and activity [16], [17]. Ucp2 activity neutralizes ROS [2]–[5] allowing continuous CPT1-promoted fatty acid oxidation and transcription of genes promoting mitochondrial proliferation (such as *Nrf1* and *Tfam* as described in the present study) enabling continuous support of the bioenergetics needs of sustained neuronal firing and synaptic plasticity [2]. This intracellular signaling should be supportive of neuronal activation and decreased vulnerability of neurons to cellular stress in any region where they are activated. Indeed, mice with enhanced *Ucp2*-expression in the hippocampus, substantia nigra, or striatum, resisted to neurodegeneration in models of epilepsy [7], Parkinson's disease [13], [18], [19], and global ischemia [20].

The current data suggests that the induction of *Ucp2* by birth -associated physiological stress enables metabolic adaptation to a switch available nutrient utilization that is critical for proper survival and development of hippocampal and other brain neurons. Whether impaired *Ucp2* induction by non-natural birth or by chemical interference could have long lasting effects on the functioning of the brain is an intriguing and potentially clinically relevant question. Our observation of adult UCP2 KO animals in open field and Y maze tests argue that long lasting effects of impaired UCP2 activity during development may in fact affect complex adult behaviors.
